# Creating an Asynchronous Curriculum for Your Emergency Medicine Residency

**DOI:** 10.15694/mep.2019.000090.1

**Published:** 2019-04-17

**Authors:** Trent She, Janice Shin-Kim, Hyunjoo Lee, Terry Li, Eric Steinberg

**Affiliations:** 1 Mount Sinai St. Luke’s and Mount Sinai West; 2 Mount Sinai Beth Israel; 3 Stony Brook University Hospital; 4 TeamHealth Special Ops West Coast

**Keywords:** medical education, asynchronous education, resident conference, resident education, FOAMEd, modular learning

## Abstract

This article was migrated. The article was marked as recommended.

Background

An asynchronous curriculum is one in which residents complete structured learning assignments outside of the traditional Emergency Medicine conference day. As educators are challenged with filling the time in the traditional didactic classroom setting with appropriate content while maintaining the interest of learners, asynchronous learning is becoming an essential component of Emergency Medicine resident curricula. While many residencies are investigating best practices to design and implement asynchronous education, relatively little guidance exists on the creation of such a curriculum.

Methods

Our goal was to create an asynchronous curriculum using only a chief resident and a core faculty member. Our module-based asynchronous curriculum was formulated based on recommendations from the Council of Emergency Medicine Residency Directors (CORD) ( Sadosty et al. 2009). We focused on using free open access medical education (FOAMEd) as primary content.

Results

Our residency program has successfully implemented an asynchronous curriculum for two years, and it is still ongoing. We achieved an assignment completion rate of 77.0% in the first year of implementation and 88.6% in our second year.

Conclusions

The creation and implementation of an asynchronous curriculum is manageable and well-received by Emergency Medicine residents.

## Introduction

The most recent update to the Program Requirements in Emergency Medicine created by the Accreditation Council of Graduate Medical Education (ACGME) requires at least five hours per week of didactic instruction and experiences and allows for 20% of this instruction to occur outside of weekly conference as “individualized interactive instruction,” also known as an asynchronous curriculum ( ACGME 2019). As modern residents increasingly use online-based resources for their point-of-care medical education, Emergency Medicine (EM) residencies have begun incorporating asynchronous education into their conference didactics. Although all EM residencies are held to the same standards by the ACGME Program Requirements, each residency has its own unique backgrounds, challenges, and idiosyncrasies. These differences are important considerations when creating an asynchronous curriculum and a residency-specific asynchronous curriculum can both strengthen existing knowledge and bridge educational gaps.

## Methods

Asynchronous education is becoming an essential component of emergency medicine resident curricula. In 2008, the CORD recommended a mix of synchronous, on-campus learning and asynchronous, off-campus learning as ideal for EM residencies ( Sadosty et al. 2009). Since each residency already had its own prescribed on-campus curriculum, the focus for most residencies was on creating a matching asynchronous curriculum for their residents. Although several examples of asynchronous curricula exist for educators to use ( Kornegay et al. 2016, Toohey et al. 2016, ALiEM 2017, Pensa et al. 2018), the need for adaptability and flexibility led the authors to formulate our own. We discuss this process in this article.

In 2017, our residency rolled out an 18-month module-based curriculum. The topics in our conference curriculum were categorized into 16 modules: Cardiology, Dermatology, Endocrine, Environmental, Gastroenterology/Surgery, Hematology/Oncology, Infectious Diseases, Neurology, Obstetrics/Gynecology, Ophthalmology, Otolaryngology, Psychiatry, Pulmonary, Renal, Toxicology, and Trauma. The amount of time spent on each module ranged from two to six weeks depending on representation of the particular topic in the American Board of Emergency Medicine Model of the Clinical Practice of Emergency Medicine, with more educational time allocated to modules that contained more information ( ABEM 2016). These 16 modules were scheduled throughout 18 months for completion; hence, in a 3-year residency, each module would be completed twice.

We intended our asynchronous curriculum to be completed in parallel with our regular on-campus conference curriculum. Each assignment was to be divided into three parts: readings of selected FOAMEd pieces, a five-question online assessment, and a 30-minute lecture during on-campus conference. We believed this setup would be best for information retention as residents would be required to recall topics twice for a particular assignment. We created 22 individual assignments ( MSBI EM 2018) in this fashion, scheduled at roughly bi-weekly intervals. Each assignment was expected to take no more than approximately two hours to complete.

The development and maintenance of our asynchronous curriculum required only a chief resident and a core faculty member. We believe that having our chief resident participate in curriculum creation is vital for several reasons. Firstly, we believe a resident has insight into the educational needs and demands of her or his co-residents. Secondly, since chief residents change annually, it allows for the curriculum to be updated annually with fresh educational content. Lastly, chief residents that are asked to take charge of the asynchronous curriculum typically have some interest in medical education and invest much time and energy to gain experience in curriculum development to further their own academic pursuits.

We initially planned to create 39 asynchronous assignments to match the 78 weeks (18 months) of on-campus curriculum. After analyzing our yearly conference schedule and accounting for the days where conference was not bound by our modular curriculum - holidays, simulation days, regional conferences, mock oral boards, etc. - we noted that we did not need 39 total assignments to cover our intended 18-month course. The final number we came to was 22 assignments to cover a roughly bi-weekly pattern over 18 months.

We chose to have our asynchronous assignments consist entirely of online FOAMEd content. Today’s residents are very familiar with FOAMEd to the point where it has become a primary method of learning, more so than previous traditional methods - journal articles, textbook readings, etc ( Mallin et al. 2014). Although FOAMEd is more accessible than these traditional sources, the quality of each piece is variable and not always peer-reviewed. Since residents are less likely to critically evaluate each piece’s source material, we needed to both search for quality content and verify its validity.

To start, we used RSS feeds and Twitter as content aggregators to search for FOAMEd pieces. We had little difficulty locating a large amount of online content. FOAMEd sources have grown tremendously and as of 2013, there are 141 blogs and 42 podcasts on emergency medicine and critical care ( Cadogan et al. 2014). In order to screen this large collection of content, the chief resident and faculty member performed literature searches and reviews on any piece marked for assignment inclusion. We were also more likely to select content from “popular” FOAMEd websites as judged by the site’s social media index - a comparative index looking at the impact of a particular EM or critical care website ( Thoma et al. 2015).

Once content was selected, the chief resident and core faculty member read through each piece and generated a five-question assessment that highlighted important takeaways in each article. Assessments were made using the Google Forms application. We selected Google Forms for several reasons - it has no fees, allows multiple people to fill out and complete a form at any given time, tracks both attendance and completion of a form, and uses the same Google platform as Google Mail and Google Calendar which were integral parts of our residency conference already. New chief residents worked with the faculty mentor to update the asynchronous assignment for the upcoming year with any new significant articles in Emergency Medicine.

To ensure retention of educational content, a 30-minute discussion was given on the asynchronous material during on-campus conference time after the assignment due date. Our faculty mentor reviewed the highlights of the assignment and elaborated on all important, relevant facts. We put our asynchronous curriculum into effect at the beginning of the academic year in 2017, which coincided with the start of an 18-month curriculum period. A total of 49 residents were part of the initial implementation and completed assignments throughout the year.

## Results

Since the implementation of our asynchronous assignments, we have had very positive feedback and a high degree of resident participation - 77.0% assignment completion rate in 2017 and 88.6% assignment completion rate in 2018. We elected to track the percentage of assignment completion rather than our residents’ scores on the five-question assessment. We believe that high assignment completion rate indicated that residents were receptive to this new approach of education and the overall score they received on the assignment was less important as a metric than the completion of the assignment itself. In future years, we intend to keep track of both resident scoring and completion percentage.

## Discussion

We made several changes to our asynchronous curriculum after its first year. We had initially decided that our 30-minute summary lecture should focus on reviewing the five questions of the assignment, but found that residents did not remember the specific questions if they completed the assessment several days to weeks beforehand. Thus, we adapted our 30-minute lecture into a summary of key takeaways from the assignment and added detailed explanation of each answers into the online assessment itself. Moreover, we chose to incorporate more clinical vignettes to the five-question assessment rather than rote memorization questions to mirror what residents may see on standardized tests. Although we strived to update a module with new FOAMEd pieces, we made a decision to repeat some particularly noteworthy pieces from prior years, especially those that discussed landmark advances in Emergency Medicine or critical care.

## Conclusion

Developing an asynchronous curriculum is certainly challenging and ours is in its third year. Our curriculum is updated and reviewed annually by our chief residents and core faculty member. The authors of this piece have greatly enjoyed the time and effort put into making our very own asynchronous curriculum. We have shared our experiences here in hope that this may help other programs launch their own curriculums and demonstrate that only necessities required to create an effective educational experience are the tenacious efforts of a dedicated few. In the spirit of medical education, please feel free to email the authors for a copy of our curriculum that may serve as the basis to your own. We have also attached a copy of our Trauma asynchronous assignment as a supplementary file to this manuscript labeled “Supplementary File 1”.

For this upcoming year, we have moved our asynchronous curriculum to an online learning management system, Schoology. By moving onto an online system, we were able to better keep track of scoring and completion rate. Furthermore, we hope to tailor our asynchronous curriculum to each resident in future after completion of the second 18-month cycle of their residency.

## Take Home Messages

• Asynchronous education is a relatively novel method of medical education which focuses on structured learning assignments outside of dedicated learning time.
• Residency programs are striving to incorporate asynchronous education into already existing curricula.
• There is a large amount of free online access medical education (FOAMEd) material that may be used to facilitate creation of an asynchronous curriculum.
• Our asynchronous curriculum required no additional funding and the support of only one chief resident and one faculty member.
• Our curriculum setup consisting of curated FOAMEd pieces, a five-question assignment, and a subsequent 30-minute lecture was well received by our residents.

## Notes On Contributors

Trent She, MD is an Emergency Ultrasound Fellow at the Department of Emergency Medicine, Mount Sinai St. Luke’s and Mount Sinai West, New York, New York. He will be staying at this institution next year as Assistant Professor of Emergency Medicine.

Janice Shin-Kim, MD is a Chief Resident at the Department of Emergency Medicine, Mount Sinai Beth Israel, New York, New York. She will be pursuing a Simulation Fellowship next year at Department of Emergency Medicine, New York University Langone Health, New York, New York.

Hyunjoo Lee, MD completed a residency in Emergency Medicine at Mount Sinai Beth Israel in New York City. She completed a fellowship in Medical Education at Thomas Jefferson University in Philadelphia and is now a Clinical Assistant Professor at Department of Emergency Medicine, Stony Brook University, Stony Brook, New York.

Terry Li, MD completed a residency in Emergency Medicine at Mount Sinai Beth Israel, New York, New York. He now works clinically for TeamHealth Special Ops West Coast, Knoxville,Tennessee.

Eric Steinberg, DO is an Assistant Professor and the Assistant Program Director at Department of Emergency Medicine, Mount Sinai Beth Israel, New York, New York. He will be taking the role of Program Director next year at Department of Emergency Medicine, St. Joseph’s Regional Medical Center, Paterson, New Jersey.
