# Supplementary material for: Creating an Asynchronous Curriculum for Your Emergency Medicine Residency
Source: MedEdPublish (2016). 2019 Apr 17;8:90. [Version 1] doi: 10.15694/mep.2019.000090.1 (PMC10712648; doi:10.15694/mep.2019.000090.1)
Supplement: Peer review history

# Reviewer report: Reviewer response for version 1

**DOI:** 10.21956/mep.19834.r29898

**Published:** 2020-03-29

**Authors:** Louise Baird

**Affiliations:** 1 St George Hospital

This review has been migrated. The reviewer awarded 2 stars out of 5 This study described the introduction of a new curricular component to the institution’s overall program in Emergency Medicine training. The methods were clear, however results were very limited as was the discussion. ‘Positive feedback’ needs further description – does this just refer to the completion rate, or did you collect qualitative feedback comments? Did the program help a higher number of residents complete the Emergency Medicine training requirements than before the program was introduced? Did the program improve resident’s work based assessments? Was there evidence their learning was enhanced by completion of the program? Your results should be compared to other similar initiatives in the published literature. Also is it feasible to update the program each year? What is the backup if these online resources become unavailable? What are the strengths and weaknesses of the study?The conclusion reads more as a reflection – try to keep the comments to scientific conclusions that reflect the research you undertook.

Reviewer Expertise:

NA

No decision status is available

# Reviewer report: Reviewer response for version 1

**DOI:** 10.21956/mep.19834.r29897

**Published:** 2019-08-26

**Authors:** Sandra Jaramillo

**Affiliations:** 1 Universidad de los Andes

This review has been migrated. The reviewer awarded 3 stars out of 5 This article is clear and pleasant to read, associated with a subject with a high impact in the area of medical education. It would be interesting to be able to know the true impact of the creation and application of the asynchronous curriculum in emergency residency program both in students and teachers beyond knowing the compliance, in terms of satisfaction, impact on the time of study and learning to give the results a more weight.It would be equally valuable to know what the causes for students who did not complete the modules and if the results are shown here must do only with the motivation and commitment of the students to the activities.As a strength, I would like to highlight the process that the authors were carried out to design the curriculum and the clear way in which the data is presented in the article.This article might be interesting for those educators interested in virtual and blended learning, not just Emergency medicine teachers.

Reviewer Expertise:

NA

No decision status is available

# Reviewer report: Reviewer response for version 1

**DOI:** 10.21956/mep.19834.r29900

**Published:** 2019-08-26

**Authors:** Simon Ling

**Affiliations:** 1 KK Women’s & Children’s Hospital

This review has been migrated. The reviewer awarded 4 stars out of 5 This article outlines the adoption of asynchronous curriculum, use of FOAMEd, faculty guidance and use of online forms for Emergency medicine. As the authors write, this is an area which programmes are struggling to incorporate.The Methods are fairly well stated. I note that CORD has recommended its use. If you had performed a Needs Assessment in order to determine your asynchronous curriculum content, then this would aid your methodology. Likewise more specific detail in how you selected FOAMEd would benefit readers in terms of implementation and strengthen your Discussion (as mentioned by other reviewers)I particularly like the follow up on-campus Faculty-facilitated reinforcement of learning - “synchronising the asynchronous” if you will. A discussion on whether the educational benefit came mostly from the asynchronous content or the on-campus teaching could be included or be a direction for further work.

Reviewer Expertise:

NA

No decision status is available

# Reviewer report: Reviewer response for version 1

**DOI:** 10.21956/mep.19834.r29899

**Published:** 2019-07-30

**Authors:** Ken Masters

**Affiliations:** 1 Sultan Qaboos University

This review has been migrated. The reviewer awarded 3 stars out of 5 The paper deals with creating an Asynchronous Curriculum for Emergency Medicine Residents. I particularly like the fact that the authors give a detailed account of the structure and use of materials (primarily open access materials from FOAMEd)Quite a bit of work needs to be done on the paper, almost all of it involving re-structuring:• Some of the material in the first paragraph of the Methods should be moved into the Introduction. (And CORD needs to be written out in full in the paper, not only in the Abstract, as does FOAMEd).• Although one can read between the lines to more or less determine the goal or aim of the project, it is never clearly stated in the paper. Although the paper is not a research paper as such, it is structured along similar lines, and so, at the end of the Introduction, it would be useful if clear and specific goal(s)/aims(s) can be stated, so that one can properly assess the extent to which these have been achieved.• Almost the entire Discussion (apart from the last sentence) should be part of the Methods, and needs to be moved into the Methods, as it describes what was done in the carrying out of the project. As a result, there really is very little remaining in the Discussion, and this is a major failing of the paper. There does need to be some reflection on the project, especially on how it meets the stated goals and aims, and also how it relates to the literature in the Introduction. (Again, not to the same extent that one would expect in a full research paper, but there does need to be something there.)• The final paragraph in the Conclusion should be at the end of the Discussion. New material should not be introduced in the Conclusion.So, a useful project, but the paper still needs some work. The project also does appear to be a useful basis for a more detailed research paper to follow, and I look forward to that paper also.

Reviewer Expertise:

NA

No decision status is available

# Reviewer report: Reviewer response for version 1

**DOI:** 10.21956/mep.19834.r29896

**Published:** 2019-04-29

**Authors:** Saee Deshpande

**Affiliations:** 1 Maharashtra University of Health Sciences, Nashik- VSPM Dental College Nagpur

This review has been migrated. The reviewer awarded 4 stars out of 5 This is a very comprehensive article with sound methodology and very well written. however, if the authors could mention what were the factors which worked in favor of this intervention, it will help those who may wish to replicate this in their settings.

Reviewer Expertise:

NA

No decision status is available

# Reviewer report: Reviewer response for version 1

**DOI:** 10.21956/mep.19834.r29894

**Published:** 2019-04-20

**Authors:** Haresh Soorma

**Affiliations:** 1 Mount Sinai

This review has been migrated. The reviewer awarded 5 stars out of 5 Very comprehensive article!

Reviewer Expertise:

NA

No decision status is available

# Reviewer report: Reviewer response for version 1

**DOI:** 10.21956/mep.19834.r29895

**Published:** 2019-04-20

**Authors:** Benjamin Laraway

**Affiliations:** 1 Mount Sinai

This review has been migrated. The reviewer awarded 5 stars out of 5 This will change how I practice.

Reviewer Expertise:

NA

No decision status is available
